# Precision non-invasive brain stimulation: an *in silico* pipeline for personalized control of brain dynamics

**DOI:** 10.1088/1741-2552/adb88f

**Published:** 2025-04-22

**Authors:** Fariba Karimi, Melanie Steiner, Taylor Newton, Bryn A Lloyd, Antonino M Cassara, Paul de Fontenay, Silvia Farcito, Jan Paul Triebkorn, Elena Beanato, Huifang Wang, Elisabetta Iavarone, Friedhelm C Hummel, Niels Kuster, Viktor Jirsa, Esra Neufeld

**Affiliations:** 1 Foundation for Research on Information Technologies in Society (IT’IS), Zurich, Switzerland; 2 Department of Information Technology and Electrical Engineering, Swiss Federal Institute of Technology (ETH), Zurich, Switzerland; 3 Institut de Neurosciences des Systémes, Marseille, France; 4 Defitech Chair of Clinical Neuroengineering, Center for Neuroprosthetics (CNP) and Brain Mind Institute (BMI), Swiss Federal Institute of Technology (EPFL) Geneva, Switzerland; 5 Defitech Chair of Clinical Neuroengineering, Center for Neuroprosthetics (CNP) and Brain Mind Institute (BMI), Swiss Federal Institute of Technology (EPFL Valais), Clinique Romande de Réadaptation, Sion, Switzerland; 6 Clinical Neuroscience, University of Geneva Medical School, Geneva, Switzerland

**Keywords:** non-invasive brain stimulation, whole brain network modeling, image-based modeling, personalized stimulation planning, response-driven neuromodulation

## Abstract

*Objective.* Non-invasive brain stimulation (NIBS) offers therapeutic benefits for various brain disorders. Personalization may enhance these benefits by optimizing stimulation parameters for individual subjects. *Approach.* We present a computational pipeline for simulating and assessing the effects of NIBS using personalized, large-scale brain network activity models. Using structural MRI and diffusion-weighted imaging data, the pipeline leverages a convolutional neural network-based segmentation algorithm to generate subject-specific head models with up to 40 tissue types and personalized dielectric properties. We integrate electromagnetic simulations of NIBS exposure with whole-brain network models to predict NIBS-dependent perturbations in brain dynamics, simulate the resulting EEG traces, and quantify metrics of brain dynamics. *Main results.* The pipeline is implemented on o^2^S^2^PARC, an open, cloud-based infrastructure designed for collaborative and reproducible computational life science. Furthermore, a dedicated planning tool provides guidance for optimizing electrode placements for transcranial temporal interference stimulation. In two proof-of-concept applications, we demonstrate that: (i) transcranial alternating current stimulation produces expected shifts in the EEG spectral response, and (ii) simulated baseline network activity exhibits physiologically plausible fluctuations in inter-hemispheric synchronization. *Significance.* This pipeline facilitates a shift from exposure-based to response-driven optimization of NIBS, supporting new stimulation paradigms that steer brain dynamics towards desired activity patterns in a controlled manner.

## 1. Introduction

### 1.1. Background

Non-invasive brain stimulation (NIBS) techniques have gained attention as treatments for neurological and psychiatric disorders [1]. These technologies offer a targeted, reversible approach to alleviate the symptoms of debilitating conditions such as stroke, dementia, chronic pain, Parkinson’s disease, traumatic brain injury and various mental health disorders [2]. In addition, methods such as transcranial magnetic stimulation (TMS) and transcranial electrical stimulation (tES) can be used to probe brain activity, helping to shed light on fundamental questions concerning the physiology of neural circuits, synaptic plasticity, and network dynamics. Emerging evidence suggests that NIBS can also enhance cognitive functions in healthy individuals [3, 4]. However, important inter-individual variability in the response to treatment strongly suggests that sophisticated, personalized interventions are required for optimal clinical outcomes—something that is laborious and often impossible to achieve through phenomenological stimulation parameter tuning. Furthermore, though interest in NIBS as a clinical tool is growing, key challenges remain, including a comprehensive elucidation of its mechanistic underpinnings, and a solution to the problem of ‘combinatorial explosion’ in the search space of stimulation parameters. These include the location, intensity, and frequency of stimulation (among others), the optimization of which is further complicated by the above-mentioned inter-individual variability of responses. Computational models can help on both fronts, clarifying the underlying interaction mechanisms of NIBS, while enhancing its efficacy by optimizing stimulation parameters and minimizing side effects. Thus, modeling can aid in the development of personalized treatments that would otherwise be challenging to achieve through experimental tuning alone. Additionally, computational models facilitate a shift from an exposure targeting paradigm to a response-driven approach that centers the effects of stimulation on neural dynamics and, ultimately, patient outcomes. Furthermore, numerical modeling promises to address inter-subject variability in the context of personalized therapy, and serves as a testing ground for new methods in the treatment of brain disorders.

Models that seek to understand the effects of neurostimulation on brain activity or to optimize stimulation delivery typically involve one or both of the following: (i) predictions of the electric field (E-field) distributions generated in brain tissues by applied electrical currents, and (ii) modeling of the neural responses elicited by these E-field distributions. In the paragraphs that follow, we discuss the history and state-of-the-art of each in turn.

Biophysical models of electromagnetic (EM) field exposure in the head and brain have undergone remarkable advances, propelled by improvements in computing power and medical imaging. The simplified head models utilized in early efforts (i.e. concentric spherical/elliptical shells) [5] were gradually expanded to include finer geometrical details [6]. Later, advances in medical imaging, especially magnetic resonance imaging (MRI) and computed tomography, helped fuel the emergence of image-based, personalized anatomical models [7–9]. The use of diffusion-weighted imaging (DWI) further enhanced model precision by enabling the incorporation of subject-specific heterogeneous and anisotropic brain tissue conductivity distributions [6, 8, 10]. In recent years, open-source NIBS simulation software libraries such as ROAST [11] and SimNIBS [12] have led to automated, registration-based routines for generating personalized volume conductor models from MRI data, relying primarily on image processing techniques such as intensity normalization, atlas-based segmentation, statistical parametric mapping, and machine learning. However, the segmentation of extra-cerebral structures from MRI is not yet mature. For example, a recent study comparing state-of-the-art skull segmentation algorithms for MRI data revealed limitations in robustness and detail in the classification of extra-cerebral tissues [13]. In this context, machine learning methods, in particular neural networks, promise a ‘sea change’ in the field of medical image segmentation [14, 15]. Efforts making use of convolutional neural networks (CNNs) have already shown promise in head tissues (e.g. Rashed *et al* [16]), though questions regarding generalization remain, particularly for MRI datasets of diverse origin and mixed scanning protocols. Moreover, these toolboxes typically segment a small number of tissue classes, or fail to provide sufficient accuracy in the scalp, skull, dura, and cerebrospinal fluid (CSF), where subtle variations in geometry are known to strongly influence predicted distributions of the E-field [9]. Our work aims to address these gaps by leveraging advanced artificial intelligence (AI)-based methods to automatically, robustly, and rapidly produce accurate segmentations of the brain and extra-cerebral structures.

Models of the neural response to electrical stimulation typically focus on one of three spatial scales—the microscale, mesoscale, or macroscale—with associated degrees of biophysical detail. At the microscale, models represent the physiology of individual neurons and their mutual interactions via synaptic connections, gap junctions, and volume transmission. While a handful of efforts have been made to upscale such highly accurate microscale models to representations of cortical microcircuitry, various subcortical structures, or even the entire rodent brain [17–19], the associated computational costs and large numbers of parameters prohibit personalization [20, 21]. At the mesoscale, models focus on intermediate descriptions of neural activity that lie between individual cells and the entire brain. Mesoscale models may represent neural circuits as connected graphs whose nodes correspond to populations of cells with simplified spiking dynamics. They help provide insights into network dynamics (often at the level of functional brain regions, or specific circuits) while reducing computational complexity [22]. Finally, macroscale models attempt to present an integrated picture of brain physiology resulting from the dynamic interplay between multiple brain regions [23]. Macroscale models are designed to relate naturally to (human) whole-brain recordings such as electroencephalography (EEG) and functional MRI (fMRI) [23], and rely on top-down descriptions of the ‘bulk’ properties of neural populations. Mesoscale neural population dynamics—bridging the gap between microscale dynamics and macroscale activity—may be represented using ‘neural mass models’ (NMMs) or ‘neural field models’ (NFMs), in which state variables such as synaptic activity and firing rate are coarse-grained in time (both NMMs and NFMs) and space (NFMs) to capture aggregate measures of activity while accounting for local interactions [24]. NMMs are rooted in the mean field formalism, which replaces a multitude of individual interactions with a single average or effective interaction while ensuring self-consistency. These models replace the behavior of an entire population with the dynamics of a single, representative neural mass, on the basis of certain statistical assumptions [25, 26]. Thus, NMMs collapse the average activity of an ensemble to a single point. Connected graphs of NMMs are a practical foundation for constructing whole brain models (WBMs), as they provide a natural, computationally efficient, parameter-reduced description of functionally distinct brain regions. The raw activity of NMMs can be translated into (electro-)physiological signals such as EEG and/or fMRI, facilitating direct comparisons with experimental data [27]. For EEG, this is achieved via lead field (LF) matrices, which map source activity in the brain to sensor recordings, and can be computed through forward modeling of E-field distributions in head and brain tissues [27]. For fMRI, the translation involves modeling the metabolic and hemodynamic response to neural activity, typically through convolution with a hemodynamic response function kernel, as implemented in platforms such as The Virtual Brain (TVB) [27]. Certain model parameters (e.g. synaptic rate constants) must be tuned against measured (electro-)physiological signals to achieve accurate and meaningful personalized outcomes [28]. Others, however, (e.g. inter-regional connection strengths) can be inferred directly from structural and functional connectivity (SC and FC) as measured using DWI and fMRI/EEG, respectively [27].

Linking dosimetric exposures to ensuing effects on brain activity requires a mechanistic or phenomenological model of the effects of fields on neurons. At the (sub-)cellular level, field-neuron coupling is a consequence of induced transmembrane and axial currents [29], membrane polarization [29], alterations in volume conduction [30, 31], and the modulation of synaptic dynamics [32, 33], among others, caused by an applied extracellular potential. While the effects of external fields on single neurons are well understood, the mechanisms of network-level effects are less clear. A majority of prior studies featuring hybrid EM—brain network activity modeling focused on deep brain stimulation (DBS), thus skirting the need for a comprehensive treatment of distributed activity in spatially embedded networks [20]. Nevertheless, Molaee-Ardekani *et al* [34] conducted a seminal study that explored the physiological impact of NIBS using a Jansen-Rit-based NMM network with a ‘lambda-E’ coupling term [35]. The ‘lambda-E’ term assumes that the E-field component normal to the cortex is responsible for linear shifts in mean membrane potential. The authors concluded that while the impact of stimulation on interneurons is not negligible, it is relatively modest compared to its effect on pyramidal cells.

Given the high dimensonality and complexity of brain dynamics, there is a need for quantitative metrics characterizing activity, state, and overall health. Following a prolonged period of over-reliance on static FC for characterizing brain state [36]), the last decade has seen increased appreciation for the importance of brain fluidity, i.e. dynamic and rapid oscillations between connectivity states, and the emergence of dynamic functional connectivity (DFC) as a tool for quantifying state changes over time [36–38]. DFC provides a broader view of spatiotemporal dynamics in brain networks, allowing for the identification of ‘meta-states’ comprising oscillatory patterns in FC. This method has been used to study various aspects of brain physiology and psychology, including epilepsy [39], sex discrimination [40], brain fingerprinting [41], and the neurological mechanisms of attention deficit hyperactivity disorder (ADHD) [42] and Alzheimer’s Disease [43], among others. Various conditions, including brain diseases and normal aging, have been found to be associated with reduced fluidity [44–47]. Thus, DFC-informed analyses can serve to extend insights offered by computational models regarding the effects of neurostimulation on brain network dynamics.

### 1.2. Key challenges

While WBMs have emerged as powerful tools to investigate brain dynamics, ongoing challenges in their implementation and use warrant discussion. One important hurdle is the inter-individual variability of structure and function, with implications for the accuracy of EM exposure calculations, brain network response modeling, and EEG signal predictions. To address this concern, WBM efforts have begun using personalized SC to inform network connection weights. Owing to recent advances in imaging technology, SC inferred from DWI data has been increasingly incorporated into WBMs [48]. Furthermore, a shift away from generic volume conductor models towards personalized anatomies has demonstrated superior accuracy for predicting field distributions. Personalized head models are increasingly used in computational studies of various neurostimulation techniques including TMS [49], tES [50], and transcranial temporal interference stimulation (tTIS) [9]. Also, LF matrices derived from personalized anatomical models have been shown to outperform those computed using generic spherical models or three-shell boundary element models [51, 52]. Last, the identification of personalized brain network model parameters informed by subject-specific brain signal measurements is still in the early stages of development [53]. At present, though researchers have begun to introduce personalization in one or more of the modeling stages described above, few efforts in the field have yet attempted a comprehensive integration of personalization throughout each step of the modeling process.

Another key limitation is the under-development of sophisticated representations of the space- and time-dependent responses to neurostimulation. A pioneering study by Spiegler *et al* [54] explored the selective activation of brain networks through simulated perturbations of different brain regions, providing a lookup table for stimulation targets and resting state networks. More recently, the same group applied a similar method in an *in silico* mouse brain model, in which they observed the emergence of known functional networks in response to virtual stimulation [55]. Various other efforts have used WBMs to explore the effects of TMS [56], repetitive TMS [57], and NIBS [58], and to study the dependence of stimulus-related network responses on personalized SC [59], network plasticity [60], and ADHD-related alterations in resting state dynamics [61]. However, many of these studies suffer from a lack of personalization, overly simplified anatomical models, and/or unrealistic representations of exposure.

Finally, while they provide a window into global network dynamics, WBMs have been underutilized as a tool for designing stimulation strategies to achieve desired brain states and improve clinical outcomes. The WBM field has focused primarily on optimizing E-field distributions in tES [62] and TMS [63]. However, this focus on field shaping neglects the important aspect of the brain dynamic response. DBS research has made strides in the use of local circuit models to optimize recruitment of a specific neural (sub-)population or functional response, but largely without considering the brain-wide systemic response [20]. A recent proof-of-concept DBS simulation study utilizing a multiscale co-simulation approach attempts to bridge a spiking model of the basal ganglia and a neural population model for cortical dynamics, but the technique has yet to be leveraged to optimize treatment protocols [64]. Currently, to the best of the authors’ knowledge, WBMs have not yet been used to optimize NIBS parameters for therapeutic purposes with the goal of shifting diseased brain networks toward healthier states.

### 1.3. Study goals

In light of the needs and challenges described above, the goals of this study were to:

• develop an integrated pipeline for constructing detailed WBMs that predict responses to space- and time-dependent neurostimulation;
• support personalized modeling of (i) anatomy, (ii) dielectric properties, (iii) SC, (iv) source-to-signal mapping (LF matrices), and (v) network dynamics;
• investigate the effects of invasive and non-invasive stimulation on network activity using state-characterizing metrics and dimensionality-reduced dynamics;
• lay groundwork for a new paradigm in which WBMs support the design of stimulation protocols and model-based closed-loop control strategies that aim to drive brain dynamics towards desirable states for improved clinical outcomes.

## 2. Methodology

**Figure 1.**
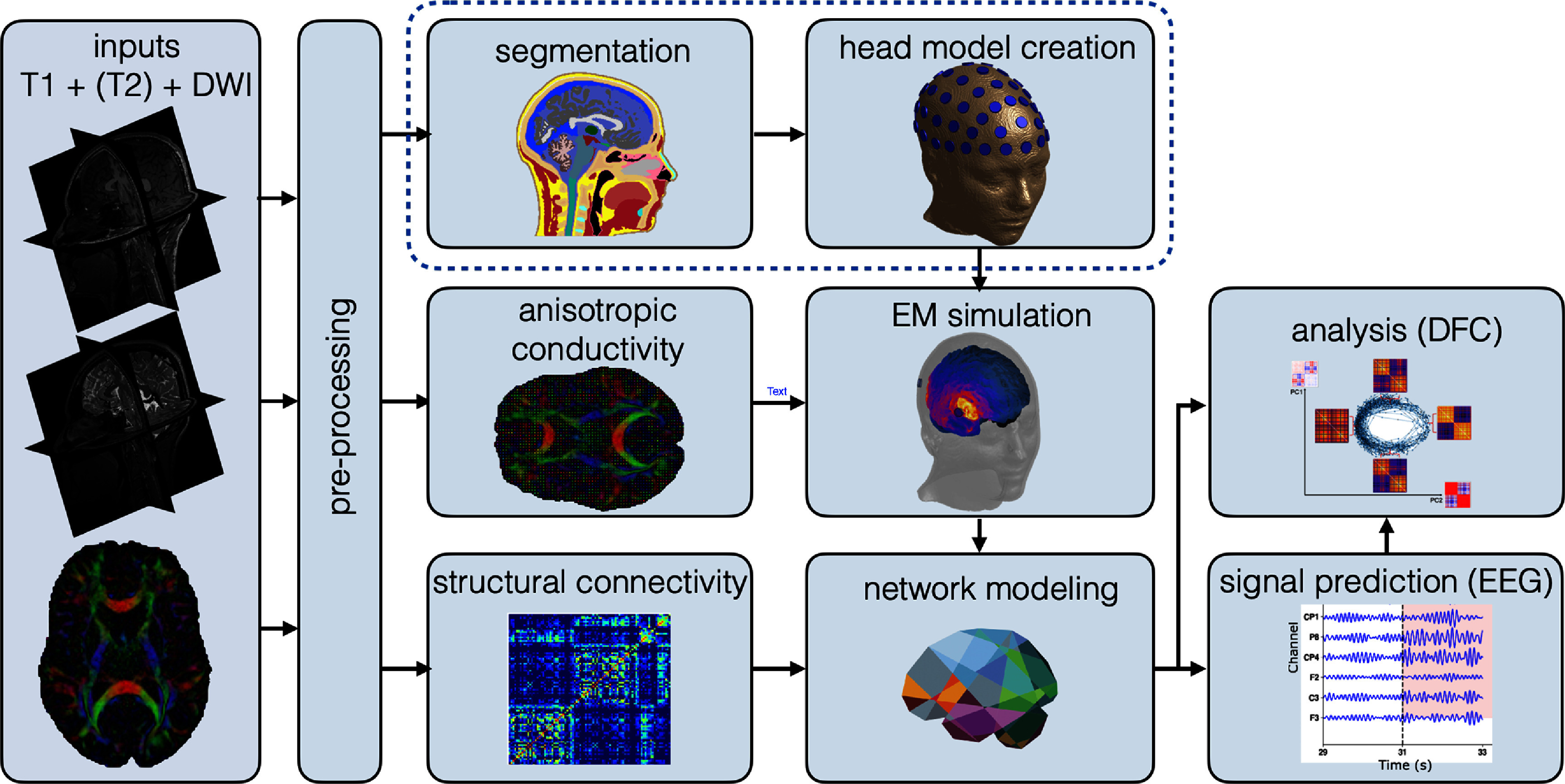
The schematic representation of the developed pipeline on o^2^S^2^PARC platform. The pipeline ingests T1-weighted MRI, optionally T2-weighted MRI, and DWI. Pre-processing includes noise reduction and co-registration of all modalities. The anisotropic conductivity values are derived from DWI and used in electromagnetic (EM) simulations. Structural connectivity (SC) matrices are obtained from DWI data and leveraged for network model personalization. EM simulations are performed to calculate exposure to stimulating E-fields and lead field matrices, which are used to predict EEG signals. Finally, higher-level network response metrics, such as dynamic functional connectivity, are quantified.

We developed a fully automated, modular pipeline for personalized modeling and simulation of the brain network response to neurostimulation (see figure 1 and table S1 in the Supplement). Starting from structural T1-/T2-weighted MRI and DWI data, the pipeline builds detailed, personalized 3D anatomical models of the head and brain, which are used to perform personalized dosimetric NIBS exposure assessments. A spatially co-registered, mean field representation of brain network activity is used to capture the dynamic response to stimulation. Finally, personalized LF matrices map source activity in the network to predicted EEG signals. Additionally, modules for a DFC-based analysis of both simulated and experimental EEG data are provided. Table S1 in the Supplement summarized of the main steps and the tools used for each module. In the following sections, we introduce the software used in the construction of the pipeline and provide a detailed explanation of each module therein.

### 2.1. o^2^S^2^PARC platform

The pipeline is implemented on o^2^S^2^PARC (osparc.io), a cloud-based, online-accessible, open-source platform for computational life science [65, 66]. This platform was developed as part of the U.S. National Institutes of Health’s Stimulating Peripheral Activity to Relieve Conditions (SPARC) program, which aims to promote FAIR (findable, accessible, interoperable, and reusable), collaborative, and sustainable computational modeling, particularly in the field of neuroscience. o^2^S^2^PARC permits users to construct computational modules, or ‘services’, from scripts, command-line tools, executables, third-party applications, neural networks, and other common elements of computational science. These services can be chained together into ‘studies’ and, with minimal effort, converted into a step-by-step, user-friendly ‘guided-mode’ format suitable for non-experts. Studies can either be directly shared for review, investigation, and modification, or made available as ‘templates’ for others to clone and adapt. When services, studies, and templates are made available publicly, they receive unique and citable identifiers suitable for use as explorable supplements in scientific publications etc. Users can access o^2^S^2^PARC through its web browser-accessible graphical user interface (GUI) or through its application programming interfaces (APIs).

### 2.2. Sim4Life

Sim4Life (ZMT Zurich MedTech AG, Switzerland), is a multiphysics simulation platform designed for computational life science. It integrates human phantoms/tissue models, high-performance computing-accelerated physics solvers, and a toolbox for computer-aided design, to support modeling of physiological and/or tissue responses, multi-goal optimization, and personalized model creation, among others. In this study, we used an o^2^S^2^PARC-hosted version of Sim4Life via its Python API to compute E-field distributions in head and brain tissues and to optimize NIBS treatment plans from an exposure point of view.

### 2.3. TVB

TVB [23, 27] is an open-source neuroinformatics platform and simulation engine for whole-brain network modeling. TVB, accessible both as a web-based GUI and as a Python library, enables the simulation and analysis of brain activity by integrating multi-modal data to generate biologically realistic brain network models, allowing for direct comparison with experimental signals. It is the principal full-brain simulator for Europe’s digital neuroscience infrastructure, EBRAINS (www.ebrains.eu). TVB natively supports a range of neural population models, and permits users to readily add/create their own [27]. Furthermore, network model personalizion facilitates the exploration of various pathological conditions and potential therapies. To constrain network connectivity, TVB provides tools for converting DWI data into SC, which is used to assign coupling weights and coupling delays to edges in the graph of network nodes (see section 2.4.1.2). TVB also offers varying levels of spatial resolution through either region-based or surface-based simulations. In the former, the brain is coarsely parcellated into regions that interact exclusively through SC. In the latter, population models are placed at each vertex of a cortical surface triangulation, allowing for higher-resolution simulations, albeit with increased computational demands [23, 27]. For this work, TVB was made available as an o^2^S^2^PARC service and integrated as a module in the pipeline.

### 2.4. Modules

#### 2.4.1. Image processing

##### 2.4.1.1. Pre-processing

The pipeline begins with the pre-processing of anatomical data, specifically the linear (affine) co-registration of all image modalities using the FMRIB Software Library (FSL)’s FLIRT [67, 68] tool. Next, using a combination of MRtrix3 [69] and FSL [70] commands, the DWI data undergoes a series of pre-processing steps including noise reduction, removal of Gibbs ringing artifacts, distortion correction, and correction for eddy current-induced artifacts. The DWI pre-processing uses existing TVB functionality [71] with minor modifications.

##### 2.4.1.2. SC extraction

SC is extracted from anatomical and DWI data using an existing TVB SC inference pipeline [71] with minor modifications. The pipeline relies on FreeSurfer for T1-weighted image pre-processing, and involves motion correction, intensity normalization, skull stripping, brain mask generation, and parcellation of cortical and subcortical regions [72, 73]. Users can select from a list of provided atlases to inform the parcellation; for this study, we chose the Desikan–Killiany atlas, comprising 84 regions (68 cortical regions and 16 subcortical regions) [74]. This is followed by comprehensive DWI pre-processing and inter-modal registration to the T1-weighted data (see section 2.4.1.1; skipped if the data was previously processed and/or already registered). Next, anatomically constrained tractography is applied to determine the location and orientation of white matter (WM) fiber tracts. These results are filtered, streamlines weights are assigned, and finally a SC matrix is constructed by aggregating the tractogram into a region-wise connectome. The steps above produce a dataset containing surface triangulations, tractography results, and SC matrices, which are used in a subsequent analysis of signal transmission between brain regions [71].

##### 2.4.1.3. Conductivity mapping from DWI

A known relationship between the water diffusion tensor and electrical conductivity [10] can be used to obtain personalized, inhomogeneous maps of anisotropic WM conductivity, which our pipeline generates in the following steps: First, diffusion tensor images (DTI) are reconstructed in the Python package DIPY [75, 76] using the pre-processed and registered DWI data, and saved in a Sim4Life-compatible format. Next, the DTI data is mapped to a conductivity tensor field in Sim4Life via the linear relationship between DTI and conductivity referred to above. The tensor fields are subsequently post-processed as follows: (i) all eigenvalues above a user-specified value (default: 2 S/m) are clamped to that value, then (ii) if any eigenvalue remains above *c* times the smallest eigenvalue, that eigenvalue is clamped to this threshold (*c* = 3 by default).

Finally, the resulting *σ* tensor and DTI tensor axes are aligned and *σ* tensor magnitudes assigned, either according to axial and radial conductivity values in the IT’IS Tissue Properties Database [77], or according to the linear relationships described in [10].

The image processing pipeline module, comprising the three steps described above (pre-processing, SC extraction, and DWI-based conductivity mapping) was implemented as a *JupyterLab* notebook-based service on o^2^S^2^PARC. The service also provides an interactive coding environment with critical libraries (FSL, FreeSurfer, and MRtrix3) pre-installed.

#### 2.4.2. Anatomical model generation

We developed a 3D U-Net-based AI segmentation tool for automated head tissue labeling in MRI data. This tool uses two sequential U-Nets: one focuses on bone prediction and the other on labeling multiple tissue classes. Our training sets included T1-/T2-weighted MRI (with manually labeled tissues) and the multi-vendor Calgary Campinas dataset (CC359). This dataset contains MRI data collected from 359 subjects using scanners from three different vendors (GE, Philips, and Siemens) operating at two distinct magnetic field strengths (1.5 T and 3 T). To improve generalization, we applied extensive data augmentation (e.g. random geometric and intensity transformations including rotation, scaling, contrast adjustment, bias field etc). Post-processing steps remove spurious clusters, fill gaps, and ensure tissue continuity. Three tool versions have been released:

• *head16*: segments 16 major tissues (released in Sim4Life V7.2).
• *head30*: segments 30 tissues using only T1-weighted MRI with minimal cleanup (Sim4Life V8.0).
• *head40*: further refines the ‘Other tissue’ class into 10 distinct classes by retraining the CNN after registering additional tissue segmentations (e.g. muscle, tendons, and glands) to the CC359 dataset.

Surfaces extracted from the label fields are then automatically smoothed and decimated using Sim4Life functionalities, making them suitable for high-fidelity anatomical modeling. The entire model generation functionality was implemented in a *JupyterLab—Sim4Life* notebook-based service on o^2^S^2^PARC, which bundles an API-accessible Sim4Life installation. For additional detail, see section S2 in the supplement.

#### 2.4.3. Electrode placement

**Figure 2.**
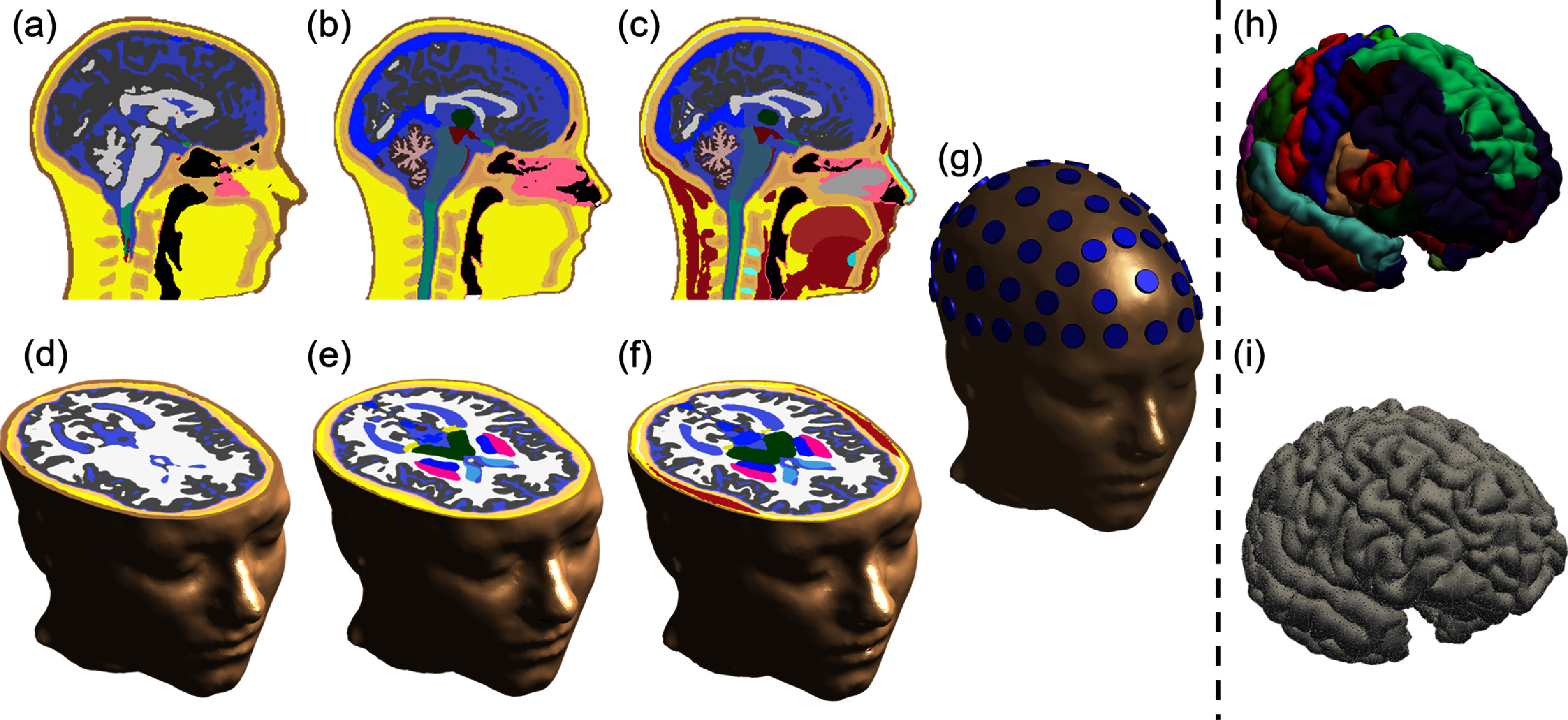
Example segmentation results for *head16* (a), *head30* (b), and *head40* (c) of AI-based approaches developed in this study and their corresponding 3D models (*head16* in (d), *head30* in (e), and *head40* in (f)). A head model featuring 10–10 EEG electrode placement is illustrated in (g). (h) Visualization of the Desikan–Killiany atlas parcellation. For region-based simulations, a neural population model is assigned to each of the parcellated regions. (i) Tessellation of the cortical surface (independent of tissue segmentation). The tessellation (shown as a mesh) represents a division of the cortical surface into fine-grained triangles. For surface-based simulations, a neural population model is assigned to each vertex of the tessellated cortical mesh. Both region- and surface-based simulations also include neural population models for specific subcortical regions.

To automate the placement of stimulating and sensing electrodes on the scalp, a dedicated tool was created that identifies the 10–10 International EEG Electrode Reference System [78] based on four anatomical landmarks: the nasion, inion, and two pre-auricular points. The reference points are identified manually in the patient-specific skin surface produced in the previous step (section 2.4.2). The tool first identifies the superior direction, the mid-sagital plane through the nasion and inion landmarks, and the transversal plane passing through the pre-auricular points. The scalp surface is then subdivided using a network of geodesic lines [79]. Finally, the tool automatically places user-defined electrode template geometries at the identified electrode positions (see figure 2(g)) by aligning the *z*-direction of the electrode with the surface normal. This step was implemented as a *JupyterLab—Sim4Life* notebook-based service on o^2^S^2^PARC.

#### 2.4.4. Dielectric property assignment

The head is a highly heterogeneous dielectric environment in which the precise assignment of dielectric properties has an important impact on exposure and recording predictions [52]. Furthermore, there is significant uncertainty and inter-subject variability associated with these properties [80]. Efforts to compile measurements of tissue dielectric properties have led to the creation of databases such as the IT’IS Tissue Properties Database [77]. This carefully curated and traceable database is derived from literature data weighted according to a range of explicit quality criteria. It compiles animal and human measurements (preferentially relying on data from large animals, such as primates, pigs, sheep, and cows) to propose average tissue properties and estimate the associated uncertainty. In the current work, most dielectric properties were assigned based on the low-frequency conductivity section of the IT’IS Tissue Properties Database V4.1. However, to account for the highly heterogeneous and anisotropic nature of brain tissue—particularly the pronounced directional dependence of electrical conductivity in WM, which can be up to ten times higher along fiber orientations than perpendicular to it—spatially-varying tensorial conductivity maps are derived from DWI data and assigned to WM regions (see section 2.4.1.3). At the frequencies of interest, dielectric dispersion can be neglected. This step was also implemented as a *JupyterLab—Sim4Life* notebook-based service on o^2^S^2^PARC.

#### 2.4.5. EM simulations

Low-frequency, electroquasistatic simulations in Sim4Life are automatically configured (in a *JupyterLab—Sim4Life* environment on o^2^S^2^PARC) and executed (leveraging o^2^S^2^PARC’s computational API for parallelized solving) to establish an E-field basis for (i) predicting brain exposures during NIBS/tES and (ii) computing EEG LF matrices to derive virtual brain-signal recordings. Since tES frequencies (1–10 kHz) are dominated by Ohmic currents, we employ Sim4Life’s electroquasistatic solver, which solves the Laplace equation for the electric potential *φ*, from which the E-field is obtained as $\mathbf{E} = -\nabla\phi$. Rectilinear (voxel-based) finite-element meshes permit to handle anatomical details robustly and in high resolution. Electrodes are modeled as floating perfect electric conductors (PEC) or Dirichlet boundary conditions, and zero-flux boundaries are applied to the head surface. The superposition principle permits pre-calculation of an E-field ‘basis’ for all electrodes, greatly reducing the required number of simulation runs. Using the reciprocity theorem [81, 82], EEG LF matrices are derived that relate intracranial dipole sources to electrode potentials. This approach significantly lowers computational cost (reducing the required simulations from *l*, the number of dipole sources, to *N*, the number of electrodes), without compromising accuracy. For additional mathematical and technical details regarding the EM simulations, see section S3 in the Supplement.

#### 2.4.6. Multi-goal exposure optimization

Our ultimate aim is to optimize the functional brain response rather than the exposure targeting per se. However, exposure optimization can serve as a valuable intermediate goal, and may also be required to establish a foundation for dynamic exposure strategies. Given the often conflicting nature of stimulation optimization objectives, we implemented a multi-objective optimization approach [83] that identifies the Pareto-optimal front of stimulation configurations. Pareto-optimal configurations are those for which it is not possible to find better performance for one objective without sacrificing performance in at least one other objective. The optimization is accomplished through grid search within the electrode placement configuration space, utilizing linear superposition of pre-computed E-field distributions from all EM simulations (see section 2.4.5). Our optimization objectives consist of the following:

• M1 - ‘strength’ (maximize): represented by the median (or *p*th isopercentile, where *p* is a user-defined value) of the quantity-of-interest (QoI) in the target region;
• M2 - ‘selectivity’ (maximize): quantified as the ratio of the mean QoI in the target region to the mean QoI in off-target regions;
• M3 - ‘collateral stimulation’ (minimize): measured as the fraction of the non-target brain volume where the QoI exceeds a specified threshold (M1).

The presence of a complete Pareto front of optimized configurations enables an interactive adjustment of the weighting of conflicting objectives in real-time [83, 84], e.g. during treatment planning in accordance with the guidance of an administering physician, or during administration in response to measured feedback. It also opens the possibility of advanced time-multiplexing schemes that rapidly switch between different optimal configurations to yield an average effect superior to any achievable with a single, static configuration [85]. The multi-goal optimization routine described above is an integrated aspect of the temporal interference planning (TIP) tool (see section 3.2).

#### 2.4.7. Brain network model generation and simulation

We employed a WBM approach based on the TVB framework, where cortical tissue is discretized into surface-based nodes. These nodes interact via short-range local connectivity (modeled by a Gaussian kernel) and long-range SC inferred from DWI data [27]. While surface-based simulations are computationally more demanding than region-based ones, they capture gyrification and support higher-resolution EEG predictions. Each node follows a Jansen-Rit NMM [86] with parameters initially assigned in accordance with the original publication (see table 1 for default values and neurophysiological analogues). Neural activity is simulated with a modified trapezoidal time integration scheme. Based on a convergence analysis comparing EEG power spectra, we selected a time step of 0.5 ms and simulation duration of 30 s, balancing numerical accuracy against computational cost. The reference spectrum was computed using a high-resolution simulation (time step: 0.01 ms, duration: 120 s). A global connectivity scale factor (*G*) was tuned to achieve dynamic richness in the simulated network FC (motivated by the observation that brain fluidity is maximal in healthy subjects). All simulations are executed in a *JupyterLab* notebook-based service on o^2^S^2^PARC. Python packages (including TVB) are preinstalled within the execution environment. For additional details, see section S4 in the supplement.

**Table 1.** Parameters of the Jansen-Rit neural mass model as described in [86], with the exception of $\mathrm{v_0}$, which is assigned the default value provided by The Virtual Brain. The table presents a detailed summary of the parameters, including their default values and physiological interpretations.

Parameter	Description	Default
*A*	Average excitatory synaptic gain	3.25 mV
*B*	Average inhibitory synaptic gain	22 mV
*a*	Time constant of excitatory post-synaptic-potential (PSP)	100 s^−1^
*b*	Time constant of inhibitory PSP	50 s^−1^
$\mathrm{\mathit C_\textit{n}}$	Average number of synaptic contacts	$\mathrm{\mathit C_1}$ = 135, $\mathrm{C_2}$ = 0.8$\mathrm{\mathit C_1}$, $\mathrm{\mathit C_3}$ = $\mathrm{\mathit C_4}$ = 0.25$\mathrm{\mathit C_1}$
$\mathrm{\mathit v_0}$	Potential at which half of the maximum firing rate is achieved	5.52 mV
$\mathrm{\mathit e_0}$	Half of the maximum firing rate	2.5 s^−1^
*r*	Steepness of the sigmoidal transformation	0.56 mV^−1^

#### 2.4.8. EM coupling to network dynamics

To represent the impact of stimulation exposure on network dynamics, we employ the $\boldsymbol{\lambda}\cdot\mathbf{E}$ model of field-neuron coupling [34, 35]. In the model, ***λ*** is the effective membrane space constant, oriented in the neuron’s orthodromic direction (assumed here to be normal to the cortex), and **E** is the local E-field. The term $\boldsymbol{\lambda}\cdot\mathbf{E}$ is incorporated as a perturbation of the mean pyramidal cell membrane potential in a given neural mass. While E-field exposure affects interneurons to some extent, its impacts are less in comparison to those on pyramidal cells [34]. Therefore, this study focuses on the effects of stimulation on pyramidal cells, omitting any influence on interneurons; however, the pipeline is designed to accommodate this influence in future investigations. Optimization of the ***λ*** coupling strength parameter is generally needed to produce accurate predictions of measurable signals (e.g. EEG) in response to stimulation. Although we provide a proof-of-concept for integrating the effects of stimulation in WBMs, validation against empirical data lies outside the scope of this study.

#### 2.4.9. Virtual EEG signal generation

After computing stimulation-dependent brain dynamics at the source level using simulations in TVB, we subsequently convert these data to sensor space (here, EEG electrode voltages) to facilitate comparisons with empirically measured data. This conversion is accomplished through multiplication with the previously computed LF matrix (see section 2.4.5).

#### 2.4.10. Metrics of network dynamics

The pipeline provides a range of analysis services that compute key metrics characterizing the dynamics of the network itself, and those of the resulting virtual EEG recordings (and other simulated (electro-)physiological signals). These services may also be used to analyze empirical measurement data, such as fMRI and EEG. In addition to providing insights into network organization, function, and dynamics, these metrics can be employed to tune and validate models against empirically measured data and to characterize brain states (and potentially brain health). Online empirical data can be used in impact-driven, closed-loop stimulation control strategies that aim to regulate/normalize brain states by driving such metrics (e.g. DFC) towards patterns or value ranges characteristic of healthy brain function. In the future, it will be desirable to complement these metrics of functional dynamics with others that shed light on network topology, information transfer, and causation, thereby offering a more comprehensive view of brain function.

DFC characterizes the dynamics of the brain’s functional connectivity based on transient FC matrices derived from source activity or sensor signals (often EEG). In our pipeline, FC matrices are computed for sliding intervals of a given duration and overlap, resulting in a temporal series of network state representations. To identify potential recurring states within the DFC patterns, k-means clustering is performed using the scikit-learn Python library [87], treating each cluster center as a network state. To enhance visualization and interpretation of the system’s dynamics, dimensionality reduction using principal component analysis (PCA) is applied. As a result, the state evolution over time can be visualized, state occurrence statistics computed, and recurring patterns in state dynamics identified. The analysis of DFC described above was implemented using an o^2^S^2^PARC-hosted *JupyterLab* notebook service that provides a suite of high-level metrics (including but not limited to DFC) that facilitate in-depth characterizations of the structural and functional organization of brain networks.

## 3. Applications and results

### 3.1. Head segmentation performance

To evaluate the performance of our AI-based segmentation tool, *head40* (and its predecessors *head30* and *head16*), we conducted a comparative analysis against two state-of-the-art methods: (i) *charm*—both the default version [88] (10 tissues and smoother labelfields) and full version (12 extra-cerebral tissues and FreeSurfer brain parcellation [72, 88, 89]) – and (ii) *headreco* [13]. Segmentation accuracy was evaluated using Dice score, Hausdorff distance (HD), mean distance (MD), and false positives (FP) [90]. These metrics show differential sensitivity to volume segmentation accuracy (overlap) and contour deviations, providing complementary information. Our assessment utilized three ground-truth datasets:

• An ‘internal dataset’ comprising six reference subjects [91]. The T1- and T2-weighted MRI data (3 T) were segmented into 16 tissue labels following the protocol established in [92]. Note that both *charm* (default/full) and *head16* were trained using the dataset developed in [92];
• The ‘MIDA head model’ [8], encompassing 115 separate tissue labels. The multi-modal dataset includes T1- and T2-weighted MRI data (3 T);
• Detailed segmentations of four subjects [93], based on the ‘IXI data’ (Guy’s Hospital, London, UK; 1.5 T) [94]. The dataset, which features 60 tissue labels, was segmented following the same conventions used to create the ‘MIDA model’ (after an appropriate mapping of dielectric properties) in terms of resolution and heterogeneity in conductivity.

Given inherent differences in the tissue labels among these datasets and the methods used for comparisons, we mapped labels to common tissue structures in both the reference and the predicted segmentations. For example, the ‘IXI dataset’ includes separate labels for mandible, skull and vertebrae, along with distinctions between cortical and cancellous tissue. For a meaningful comparison with *headreco*, which only segments ‘bone’, the individual bone structures (cortical and cancellous bone) were merged. Similarly, we combined all cortical bone labels to assess accuracy in cortical bone, and all GM deep brain structures to measure accuracy in brain GM.

The mean Dice scores are listed in tables 2–4 for the ‘internal dataset’, ‘MIDA head model’ and ‘IXI dataset’, respectively. Additional metrics including HD, MD, and FP for all datasets are provided in the supplementary materials, tables S3–S11. Across all three datasets, *head30*/*head40* demonstrate superior performance for most tissues compared to the other methods. For the ‘internal dataset’ only the mucosa has a *head30*/*head40* Dice score inferior to that of *charm (full)*. The mucosa in this fast protocol [92] is segmented only in the nasal region (same as *charm (full)* and *head16*), whereas *head30* ensures the airways are lined by a thin mucosa layer, which we consider more accurate both anatomically and in terms of material properties. For the ‘IXI dataset’, *head30*/*head40* generally outperform the other methods. In certain tissues (e.g. arteries and veins), segmentation performance appears relatively low for all three methods. In the ‘IXI’ and ‘MIDA’ datasets, this is likely attributable to a higher level of detail in the manual reference segmentation, facilitated by the availability of MR angiography data. This hypothesis is supported by FP scores for arteries and veins, which is generally favorable. For example, artery/vein FP scores in the ‘MIDA dataset’ are 0.08%, 0.01%, and 0.06% for *charm (full)*, *head16*, and *head30*, respectively. Table 5 compares the performance of *charm (full)* and *head30* for deep brain structures. For the ‘IXI dataset’, MD is reported in table 6. For all datasets, we observed superior accuracy for *head30*/*head40* as compared to the other methods. In particular, *head30*/*head40* significantly outperforms *charm (full)* for structures in the neck (spinal cord) and face (eyes, optic nerve, ocular muscle, and mandible). This difference likely results from the failure of *charm (full)* registration approach (SAMSEG) to accurately capture variations between the subject’s anatomy and the template head anatomy or neck pose.

**Table 2.** Comparison of mean Dice scores based on the ‘internal dataset’ [91]. The reference images are segmented into 15 labels, following the same protocol as in [92]. The mean Dice score for the five subjects is listed for *headreco*, *charm (full)*, *head16*, *head30*, and *head40*. The highest mean Dice scores for each label are highlighted in black, while the other values are shown in gray. ‘Bone’ is the union of cortical and cancellous bone (*headreco* does not distinguish these).

	headreco	charm	charm	head16	head30	head40
		(default)	(full)			
Air			0.589	0.649	0.681	0.666
Bone	0.760	0.796	0.796	0.809	0.812	0.816
Cortical bone		0.669	0.675	0.660	0.677	0.685
Cancellous bone		0.377	0.373	0.506	0.535	0.412
CSF	0.598	0.628	0.655	0.611	0.646	0.635
Brain GM	0.769	0.778	0.803	0.807	0.811	0.817
Brain WM	0.830	0.814	0.861	0.859	0.878	0.877
Ocular muscle		0.374	0.397	0.142	0.382	0.443
Optic nerve			0.499	0.296	0.511	0.559
Spinal cord			0.468	0.242	0.841	0.888
Artery			0.202	0.185	0.303	0.344
Eyes	0.890	0.902	0.910	0.908	0.914	0.929
Mucosa			0.702	0.440	0.580	0.519
Skin	0.240		0.641	0.683	0.747	0.737
Vein			0.496	0.529	0.429	0.441

**Table 3.** Comparison of mean Dice scores using the ‘MIDA head’ model as the gold standard. This comparison evaluates segmentation accuracy for various approaches across multiple tissue labels, with the ‘MIDA head’ model serving as the reference. The highest mean Dice scores for each label are highlighted in black, while the others are shown in gray.

	headreco	charm	charm	head16	head30	head40
		(default)	(full)			
Air	0.305		0.538	0.381	0.556	0.552
Bone	0.782	0.778	0.769	0.712	0.765	0.796
Skull					0.763	0.798
Vertebrae					0.783	0.773
CSF	0.592	0.553	0.614	0.522	0.643	0.661
Brain GM	0.817	0.819	0.814	0.718	0.785	0.813
Brain WM	0.873	0.865	0.868	0.844	0.873	0.885
Ocular muscle		0.325	0.345	0.053	0.409	0.484
Optic nerve			0.321	0.005	0.616	0.682
Spinal cord			0.815	0.646	0.879	0.878
Artery			0.280	0.013	0.280	0.302
Eyes	0.865	0.657	0.661	0.678	0.846	0.872
Mucosa			0.417	0.195	0.405	0.428
Skin	0.146		0.476	0.459	0.685	0.690
Vein			0.431	0.331	0.458	0.436

**Table 4.** Comparison of mean Dice scores based on the ‘IXI dataset’. The table lists the mean Dice score per tissue averaged across the four subjects. Note that the manual reference segmentations include 60 separate tissue labels. The highest mean Dice scores for each label are highlighted in black, while the others are shown in gray.

	headreco	charm	charm	head16	head30	head40
		(default)	(full)			
Air	0.256		0.634	0.604	0.743	0.732
Bone	0.671	0.678	0.691	0.788	0.804	0.811
Cortical bone		0.556	0.568	0.599	0.666	0.677
Cancellous bone		0.295	0.322	0.476	0.517	0.439
CSF	0.623	0.520	0.540	0.669	0.658	0.659
Brain GM	0.796	0.737	0.734	0.793	0.839	0.848
Brain WM	0.851	0.836	0.836	0.839	0.894	0.897
Optic nerve			0.390	0.346	0.568	0.589
Spinal cord			0.695	0.757	0.929	0.923
Artery			0.117	0.002	0.145	0.134
Eyes	0.844	0.754	0.750	0.851	0.922	0.931
Mucosa			0.434	0.301	0.572	0.552
Skin	0.225		0.612	0.666	0.729	0.711
Vein			0.426	0.097	0.578	0.556

**Table 5.** Performance comparison for deep brain structures measured by Dice score. Only *charm (full)* and *head30*/*head40* are compared since *headreco* and *head16* do not segment these tissue structures. The highest mean Dice scores for each label are highlighted in black, while the others are shown in gray.

	IXI		MIDA	
	charm (full)	head30/head40	charm (full)	head30/head40
Amygdala	0.749	0.888 / 0.882	0.743	0.770 / 0.762
Caudate nucleus	0.785	0.884 / 0.893	0.789	0.763 / 0.751
Globus pallidus	0.784	0.914 / 0.912	0.621	0.595 / 0.603
Hippocampus	0.828	0.887 / 0.902	0.613	0.632 / 0.662
Nucleus accumbens	0.605	0.866 / 0.855	0.642	0.646 / 0.631
Putamen	0.812	0.921 / 0.921	0.850	0.853 / 0.853
Thalamus	0.799	0.826 / 0.832	0.850	0.757 / 0.879

**Table 6.** Mean distance (MD) for the ‘IXI dataset’. For most tissues, *head30* and *head40* exhibit comparable performance, both outperforming the *charm (full/default)* and *headreco* models, consistent with observations from the other datasets. The best MD scores for each label are highlighted in black, while the others are shown in gray.

	headreco	charm	charm	head16	head30	head40
		(default)	(full)			
Air	15.540		1.863	4.213	0.747	1.692
Bone	0.734	0.845	0.714	0.249	0.203	0.204
Cortical bone		0.880	0.702	0.399	0.321	0.318
Cancellous bone		3.377	2.771	1.541	1.086	2.776
CSF	0.555	1.264	0.765	0.352	0.364	0.366
Brain GM	0.305	0.429	0.392	0.355	0.105	0.099
Brain WM	0.195	0.426	0.182	0.204	0.086	0.081
Optic nerve			1.761	4.133	0.982	0.426
Spinal cord			3.081	0.926	0.046	0.058
Artery			8.601	22.542	8.968	9.060
Eyes	0.183	1.191	1.204	0.171	0.049	0.038
Mucosa			5.074	3.909	0.514	0.504
Skin	6.853		0.992	0.321	0.226	0.234
Vein			2.912	21.645	1.466	1.549

For *head40* (and *head30*/*head16*), tissue label predictions typically require 3–5 min. Most of this time is spent in post-processing, while the inference itself takes less than a minute. By comparison, *headreco* requires nearly one hour, and *charm (default/full)* takes approximately half an hour (pure segmentation time, meshing excluded).

### 3.2. TIP tool

Identifying tTIS parameters that target a (deep) brain region of interest with maximal selectivity, while simultaneously avoiding unwanted collateral exposure and ensuring sufficient stimulation strength, is a formidable computational challenge. As a further demonstration of the power and flexibility of our methodology for creating modular computational biophysics pipelines (see section 2), we implemented a modeling and simulation tool dedicated to supporting tTIS research and addressing the challenge of identifying optimal parameters. The ‘TIP tool’ – a step-by-step application leveraging the o^2^S^2^PARC services described in section 2 and o^2^S^2^PARC’s ‘guided-mode’ application interface—facilitates use by tTIS researchers without strong modeling expertise. The primary goal of this tool is to guide the optimal placement of electrodes and selection of currents to target a brain region of interest. The following three steps constitute the core of TIP:

• Setup: definition/restriction of the stimulation parameter search space and specification of optimization parameters;
• Interactive stimulation planning: execution of the optimization routine and identification of a superior stimulation strategy by interactively weighing conflicting goals and visualizing/quantifying key exposure and performance metrics;
• Exposure analysis: detailed and flexible examination of the field distributions for the selected setup using a collection of algorithms and visualization methods available in Sim4Life.

To avoid calculating E-fields for each permutation, pre-computed E-field bases (see section 2.4.5) are utilized. TIP supports assessments based on two approaches:

(i) a library of pre-determined E-field bases for various anatomical templates, including the MIDA model ([8], isotropic and anisotropic brain conductivity variants), the IXI model (male and female, anisotropic), and a 3-week-old male mouse model [95]; and (ii) personalized E-field bases generated from user-provided MRI data using the methodologies described in sections 2.4.1–2.4.5 for personalized tTIS optimization. The fields for each channel pair of interest are then computed using the superposition principle (see section 2.4.5), and the total field for classical tTIS (two channels, sinusoidal current) is obtained as:

\begin{align*} \mathbf{E}\left(t,\mathbf{r}\right) = \sin{\left(\omega_{1} \cdot t\right)}\mathbf{E_{1}}\left(\mathbf{r}\right) + \sin{\left(\omega_{2} \cdot t\right)}\mathbf{E_{2}}\left(\mathbf{r}\right)\end{align*}

where $\mathbf{E}_{1,2}$ are the fields of the two channels independently and $\omega_{1,2}$ are their angular frequencies, where the initial phases are set to zero without loss of generality. The modulation envelope magnitude (MEM) is used to characterize tTIS exposure, computed according to the formula described in [96]. To assess the optimality of a tTIS exposure condition, three key performance metrics are considered: (i) exposure magnitude, (ii) selectivity, and (iii) avoidance of collateral stimulation, which are defined based on MEM in section 2.4.6.

#### 3.2.1. Setup

**Figure 3.**
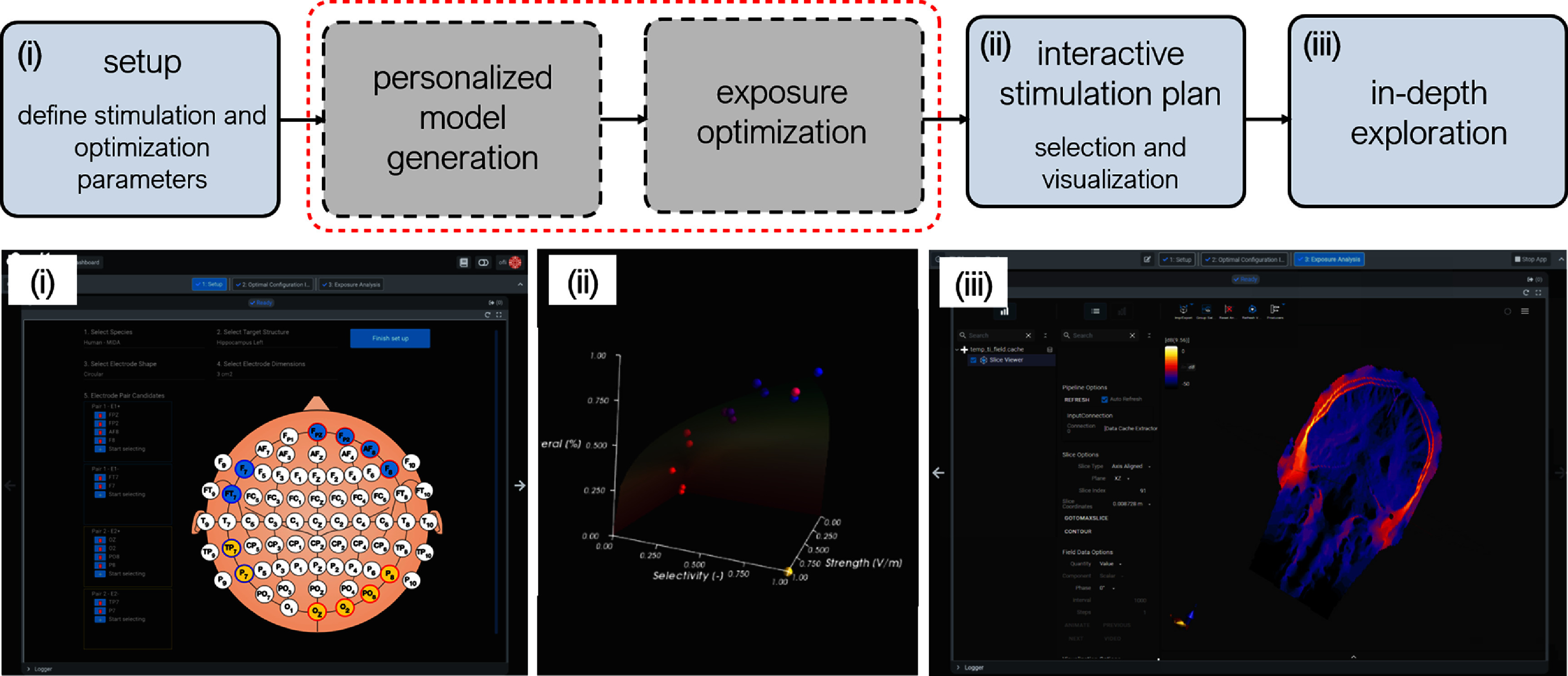
The temporal interference planning (TIP) workflow includes three interactive steps: (i) setup, (ii) interactive stimulation planning, and (iii) exposure analysis. Additionally, the pipeline includes computational services for image-based personalized model generation (including EM simulation; this service is only used for personalized temporal interference planning) and multi-goal exposure optimization.

The first step of TIP for classic tTIS (two channels, sinusoidal currents) begins with the ‘Electrode Selector’ interface, which provides users with an interactive widget to select candidate locations according to the 10–10 system for the placement of electrode pairs (search-space definition). Users can select a species of interest (currently available: human/mouse), target brain structure (from a co-registered brain atlas), electrode shape (currently available: circular), and electrode dimensions (currently available: 3 cm^2^) (see figure 3).

#### 3.2.2. Interactive stimulation planning

To determine the optimal electrode configuration for selectively targeting the identified brain region, we apply the optimization routine described in section 2.4.6, considering the three performance metrics detailed above. TIP’s dynamic weighting feature facilitates the generation of a sorted list of tTIS configurations, with a consistent prioritization of Pareto-optimal configurations. Multi-goal optimization techniques empower users to make informed decisions when confronted with conflicting tTIS exposure criteria.

Starting from an optimized or user-defined tTIS configuration, users can further explore the resulting exposure. The configuration, including electrode locations and current magnitudes, may be refined interactively. Visualization of tTIS and high frequency exposure distributions overlaid on medical imaging data, along with exposure metrics and plots, supports this refinement process. Upon identifying intended exposure conditions, associated visualizations and performance metrics can be incorporated into an automatically generated, downloadable report. tTIS has been generalized to utilize more than two channels (e.g. multi-channel tTIS for improved focality [97]) and non-sinusoidal carriers (e.g. phase-modulation to produce a wide range of envelope/pulse shapes). TIP also supports multi-channel and phase-modulated TI planning through workflow variants with adapted settings and generalized performance metrics. However, these variants limit the analysis to user-specified configurations, as systematic optimization in the vast parameter space of generalized tTIS is currently impractical.

#### 3.2.3. In-depth exposure analysis

The third step of TIP leverages the comprehensive visualization and analysis capabilities of Sim4Life (see figure 3). This enables users to review the fields produced by their selected setup. Examples of the enhanced features include volume rendering, streamline extraction, masking, projection, and the calculation of derived quantities, among others.

### 3.3. Relevance of personalized modeling

The developed pipeline (figure 1) supports personalized modeling. While emerging research increasingly draws attention to the importance of inter-individual variation in brain physiology [98], the precise role of such variation, its context-dependent relevance, and the level of detail/realism required for meaningful modeling results are still unclear. In this section, we present preliminary results obtained by applying the personalized EM modeling pipeline to data from a previous trial concerning the influence of tTIS on motor-learning in healthy subjects (*n* = 15) [91]. In that study, TIP was used to identify optimal electrode montages and stimulation parameters to selectively target the striatum. The optimal electrode locations were chosen based on precomputed E-fields obtained using the MIDA model (i.e. no personalization of head/brain anatomical models). However, once optimal electrode locations had been identified, E-field, MEM, and current density distributions were computed using personalized EM simulations. These simulations were conducted in models created using T1-/T2-weighted and DWI data, ensuring that the final exposure metrics were tailored to the specific anatomical characteristics of each participant.

#### 3.3.1. Impact of model personalization and anatomical detail on exposure predictions

To better understand the respective impacts of key aspects of the head model generation process on EM exposure predictions, we systematically compared variants of each, examining the results in terms of the optimization metrics defined in section 2.4.6. Specifically, we assessed the influence of segmentation algorithm, comparing *headreco* [13] (6 tissues classes), *charm (full)* [88] (15 tissue classes), and *head40* (see section 2.4.2; 40 tissue classes) with and without DWI-based anisotropic conductivity tensor modeling. Note that the same electrode locations—optimized using pre-computed E-fields—were used for all models, but personalized anatomical models were employed to predict tTIS exposure metrics. Additionally, both personalized and non-personalized current steering was investigated for the tTIS channels.

**Figure 4.**
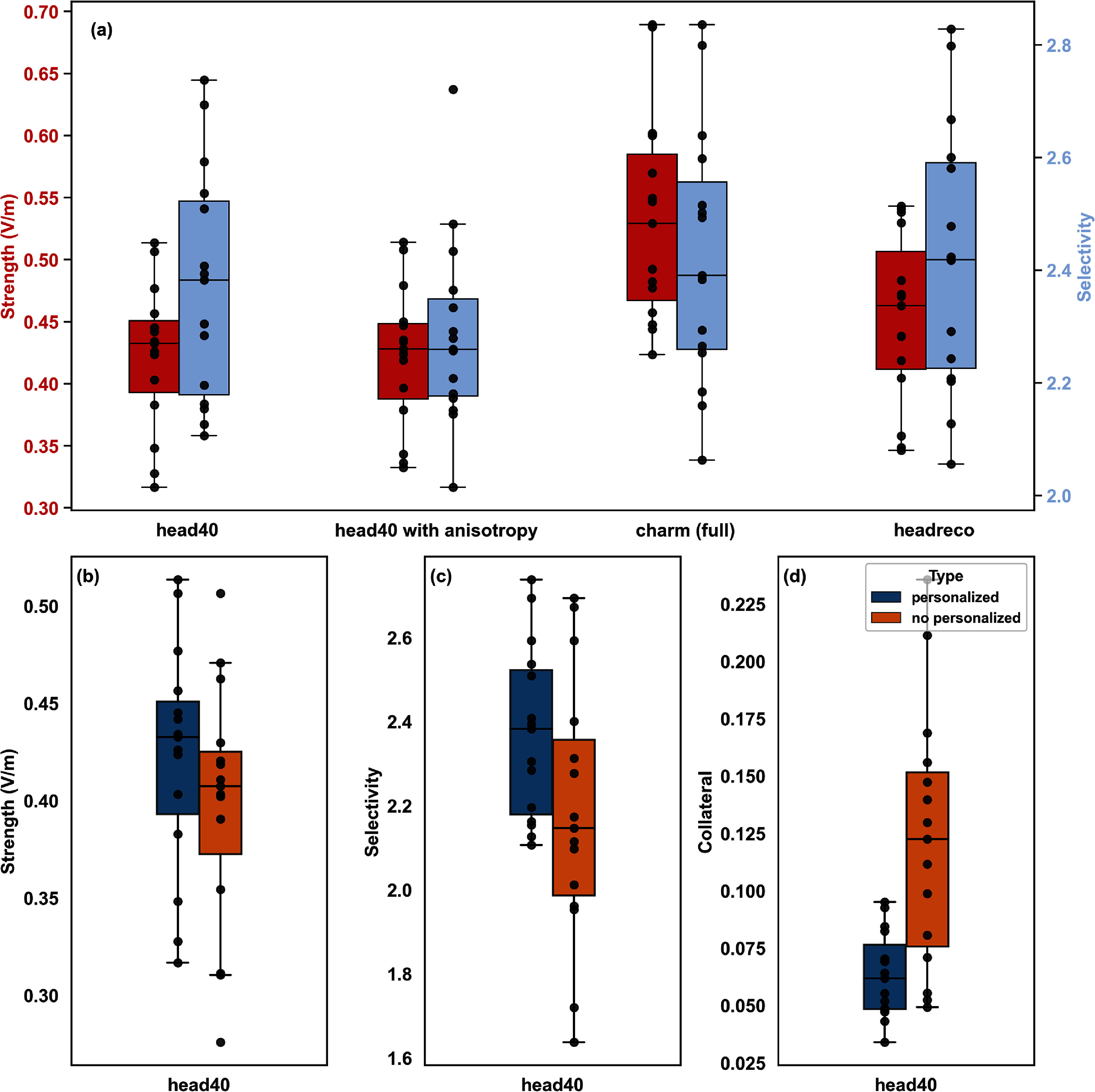
(a) Impact of model creation on the inter-subject variability (*n* = 15) of predicted performance metrics. The *X*-axis represents different segmentation conditions: *head40*, *head40* with anisotropic conductivity modeling, *charm (full)*, and *headreco*. The left *Y*-axis shows strength at the target location (striatum) as the median exposure magnitude, while the right *Y*-axis shows selectivity (ratio of target exposure to off-target exposure); (bottom) benefit of personalized current steering in terms of (b) strength, (c) selectivity, and (d) collateral exposure. In all panels, individual data points are plotted to illustrate variability, and statistical comparisons between conditions are highlighted where applicable.

Figure 4(a) compares the strength (median target MEM) and selectivity predictions for the 15 subjects across these modeling variants using personalized current steering. Inter-subject coefficient of variation in strength and selectivity is approximately 15% and 10%, respectively. Exposure metrics varied by up to a factor of four between subjects, with particularly large variability in off-target collateral exposure. The choice of segmentation algorithm had a substantial impact on exposure predictions (see figure 4(a)). To assess the statistical significance of segmentation method and personalization-related differences, we conducted both within-group and across-group repeated-measures analysis of variance (ANOVA). First, a Shapiro-Wilk test was used to confirm the normality of each dataset. Next, a within-group, repeated-measures one-way ANOVA was performed once with and once without personalized steering, to determine whether predicted exposure metrics differ significantly between the model generation conditions (*head40*, *head40* + anisotropy, *charm*, *headreco*). The one-way ANOVA for the personalized steering group revealed a highly significant segmentation-dependent off-target exposure effect ($F = 31.93, {p} < 0.0001$), indicating that the model generation approach systematically and substantially alters off-target exposure estimates. No significant segmentation effect was observed in the non-personalized steering group for collateral exposure ($F = 0.86, {p} = 0.47$). Both median exposure and selectivity demonstrated significant main effects of segmentation for the personalized and the non-personalized steering groups (${p} < 0.0001$), but the pattern of pairwise differences varied by measure (see table S12 in the supplementary materials).

Post-hoc pairwise Student’s *t*-tests with Bonferroni corrections for multiple comparisons were computed for each condition combination, and effect sizes for each were calculated using Cohen’s *d*. Large effect sizes (Cohen’s *d* > 1.0) were observed for many of the condition comparisons in the personalized group, and some in the non-personalized group, underscoring the importance of design decisions regarding anatomical segmentation (see tables S13 and S14 in the Supplementary Materials).

Next, we performed an across-group 2×4 repeated-measures ANOVA that treated steering personalization and the four model creation conditions as within-subject factors to examine the influence of personalization (type), segmentation algorithm (condition), and type×condition interactions. Collateral exposure exhibited significant main effects of both type and condition (*p* $ < 0.001$), as well as a significant type×condition interaction (*p* $ < 0.001$). This indicates that personalization strongly influences off-target exposure and that the impact of the segmentation algorithm is markedly different depending on whether the model is personalized. By contrast, while median exposure and selectivity also exhibited highly significant main effects of type and condition (*p* < 0.001), no significant interaction was found. Therefore, though personalization and segmentation algorithm each have substantial, independent impacts on the magnitude of median target exposure or selectivity, the effects of personalization are relatively consistent across segmentation algorithms for these measures. For a numerical summary of the statistics described above, see table S15 in the Supplementary Materials.

We also looked at segmentation algorithm-related E-field magnitude differences, finding that the average field strength predictions produced by *charm (full)* were 26% higher than those of *head40*. Furthermore, on average, the inclusion of anisotropic WM conductivity reduced selectivity predictions by 5% across models. However, in a complementary analysis of inter-segmentation method correlations for the personalized models, we saw that despite such differences in field strength, there was a robust correspondence between the predictions of each head model (see figure S1 in the supplement). The Pearson correlation coefficients between exposure metrics for all 15 subjects ranged from 0.67 to 1.00 for strength at the target (with only *charm (full)* correlations below 0.94), from 0.76 to 0.99 for selectivity (only models with anisotropy exhibited correlations $ < $0.95), and from 0.81 to 1.00 for collateral exposure. These high correlation values indicate consistency among the modeling approaches, suggesting that despite magnitude differences between metrics and some permutations in the subject-specific relative rankings, all make similar predictions regarding the relative impact of anatomical variation on performance metrics.

#### 3.3.2. Benefits of personalized optimization

Personalized current steering (i.e. optimizing the distribution of current across the two channels) can substantially improve exposure performance metrics—particularly the collateral exposure—as shown in figures 4(b)–(d) for the *head40* models. Personalized current steering produced an increase in strength by 6.2±0.6% (mean±std.dev. across subjects), an increase in selectivity by 9.3±1.0%, and a decrease in collateral exposure by 43.7±6.8%. The results of the 2×4 ANOVA confirm that personalization, in general, contributes strongly to improved performance metrics (main effect of type), but the magnitude of this impact is measure-dependent (see table S15). Overall, these findings underscore the practical value of individualized modeling and optimization strategies for transcranial stimulation.

### 3.4. WBM results

#### 3.4.1. DFC

**Figure 5.**
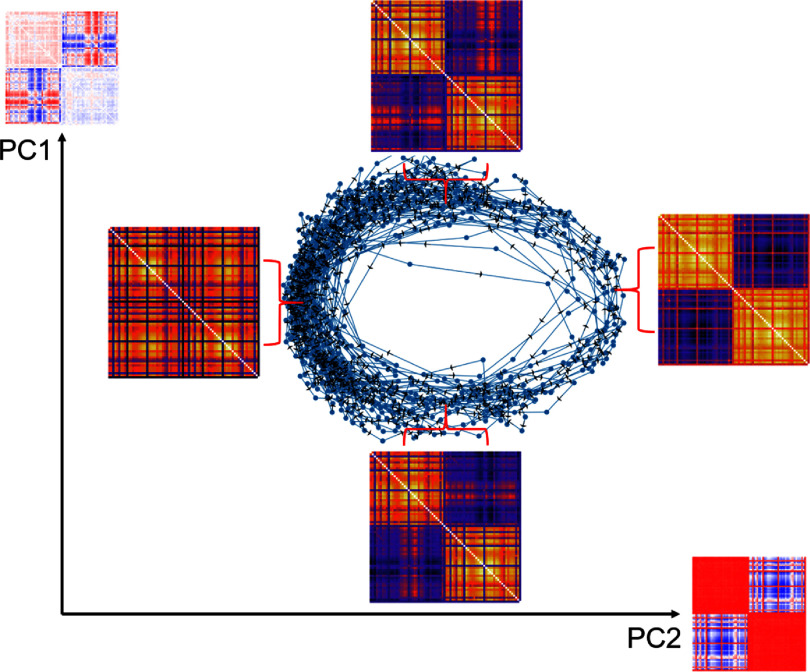
Temporal evolution of dynamic functional connectivity (DFC) in the principal component (PC) domain (two PCs are shown). Individual functional connectivity (FC) matrices associated with key clusters in PC-space are presented with arrows indicating the temporal evolution of the simulated dynamics. The first and second PCs are displayed next to their respective axes. The first PC (PC1) measures hemispheric isolation and intra-hemispheric synchrony, while the second PC (PC2) reflects the the degree of inter-hemispheric communication, particularly emphasizing the lateralized initiation of synchronization.

The Jansen-Rit model demonstrates different dynamical behaviors depending on its parameters, spanning noisy fluctuations to periodic oscillations [99]. The frequency with which brain dynamics transition between different FC regimes is sometimes referred to as ‘fluidity’, and high fluidity is a hallmark of healthy brain dynamics. To illustrate how dynamical properties in the oscillatory regime can be analyzed and described, we adjusted the inhibitory and excitatory time constants in the model to obtain a regime with fluid network dynamics (*a* = 60 s^−1^ and *b* = 30 s^−1^). Using these parameters, a region-based simulation was conducted with default SC excluding subcortical regions. The default TVB SC matrix is a bi-hemispheric hybrid that integrates Macaque connectivity data from CoCoMac-an average macro-connectome derived from over 4000 published experimental findings across approximately 250 experiments [100]-and colossal connections inferred from diffusion spectrum imaging [101], with fiber bundle widths scaled to CoCoMac’s 0-3 strength scale. The simulation duration was 40 min with a time step of 0.5 ms. Global connectivity was set at 7, and the noise level at $1\times 10^{-7}$. The Jansen-Rit parameters (except a and b) were maintained at their default values (see table 1). DFC of pyramidal cell output was computed using sliding 10 s time windows with 50% overlap. Following the calculation of DFC, PCA was applied to reduce dimensionality and facilitate visualization. The temporal evolution of DFC projected along the two principal components is depicted in figure 5. Additionally, the cluster centers of the FC matrices are illustrated in figure 5. The first and second principal components (PCs), along with their corresponding axes, are also presented.

The two PCs have a straightforward interpretation: PC2 reflects the degree of synchronization between the two hemispheres, while PC1 distinguishes initiation of synchronization/desynchronization by the left vs. right hemisphere. The simulated network dynamics mostly fluctuates around a hemisphere-synchronized state, but occasionally—initiated by either the left or the right hemisphere—desynchronization occurs, until synchronization is rescued by one of the hemispheres. The dynamics in the sub-manifold spanned by these two components is topologically equivalent to a circle, such that it can be represented by a uni-dimensional phase angle. Performing a similar analysis using subject-specific SC data (not shown here) results in similar, albeit ‘noisier’ dynamics—i.e. the PCs and dynamic behavior are qualitatively similar, but the clean circular dynamics with core-avoidant trajectories are absent, meaning that re-initiation of hemisphere synchronization cannot always be attributed clearly to one side. It is assumed that the CoCoMac-based results present an unrealistically ideal, but still representative dynamics. Similar fluctuations have been observed empirically, and typically occur on a timescale of 100 ms to seconds in EEG recordings [102] and minutes in real-world fMRI data [37].

#### 3.4.2. EEG modeling

**Figure 6.**
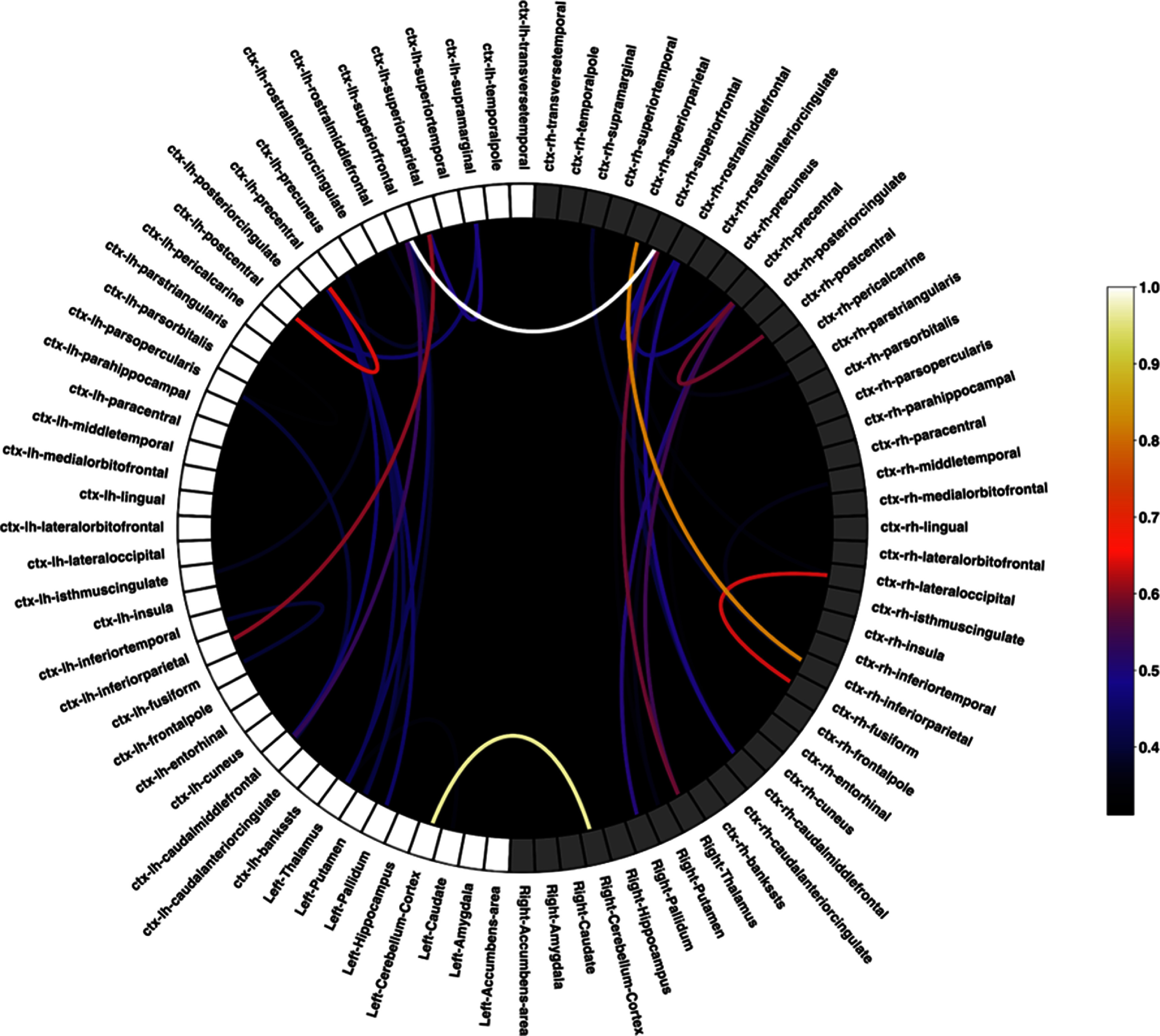
Structural connectivity (SC) illustration of of a healthy subject in a circular layout. Each node corresponds to a brain region based on the Desikan–Killiany atlas. The nodes are ordered along the circumference; white nodes represent regions in the left hemisphere and gray nodes represent regions in the right hemisphere. Cortical regions are located at the top and subcortical ones at the bottom. The lines show the strongest 20 connections (color-coded) between brain regions.

**Figure 7.**
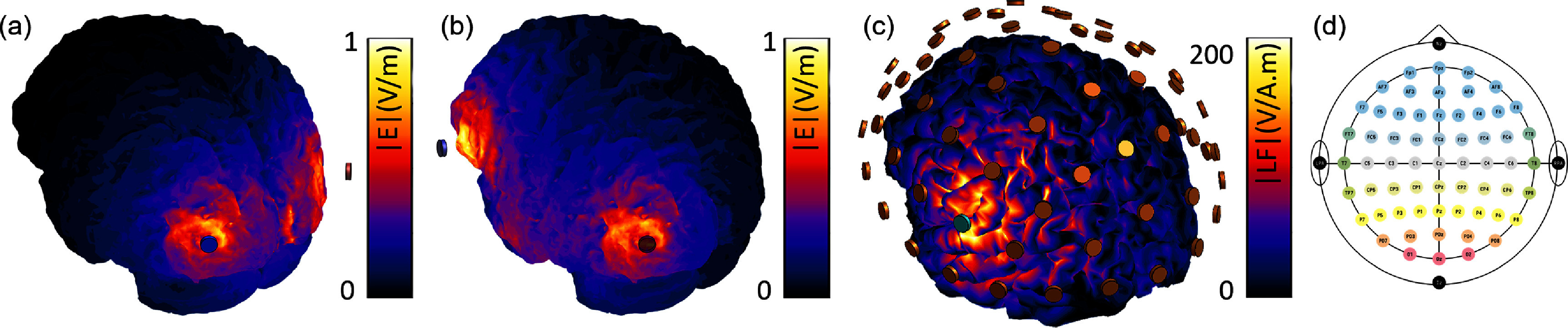
Cortical E-field distribution induced by transcranial alternating current stimulation applied through (a) P7-P8, or (b) P7-AF7 electrodes (10–10 EEG system). (c) Visualization of a lead field matrix row (corresponding to the FC5 electrode, colored in light blue). (d) 10–10 EEG system representation. This 10–10 EEG system has been adapted by the author(s) from the Wikimedia website, where it is stated to have been released into the public domain. It is included within this article on that basis [103].

To illustrate application of the complete pipeline, T1- and T2-weighted MRI data and DWI from a healthy subject were used to construct, simulate, and analyze a personalized volume conductor model of the head/brain and its physiological response to stimulation, as described in sections 2.4.1.2–2.4.9. The outcomes of various steps (SC inference, 10–10 system electrode placement, and predicted stimulation exposure) can be seen in figures 6 and 7. 2 mA stimulation currents were applied in three different transcranial alternating current stimulation (tACS) montages (P7-P8, P7-AF7, and AF7-AF8) at 8 and 15 Hz. The WBM was simulated in a surface-based configuration (∼20’000 nodes) using the Jansen-Rit model with default parameters (see table 1) for a duration of 91 s, discarding the first second to exclude transitory dynamics. A simulation duration of at least 30 s per condition was used, based on a convergence analysis, to ensure stable and reliable results. Stimulation was applied during the central 30 s of the total duration. The default parameters of the Jansen-Rit model generate dynamics within the noisy regime, effectively replicating the qualitative characteristics of EEG *α*-band activity. The time step, global connectivity scaling, and noise level were set to the same values used for the DFC modeling in section 3.4.1, namely, *G* = 7, time step = 0.5 ms, and noise level $= 1\times 10^{-7}$, while local connectivity was modeled with a Gaussian kernel (width: 10 mm, amplitude: 1, cutoff: 20 mm). The *λ* stimulation coupling parameter was adjusted to yield stimulation-related perturbations of a realistic magnitude—in practical applications, this parameter must be tuned against empirically measured data.

**Figure 8.**
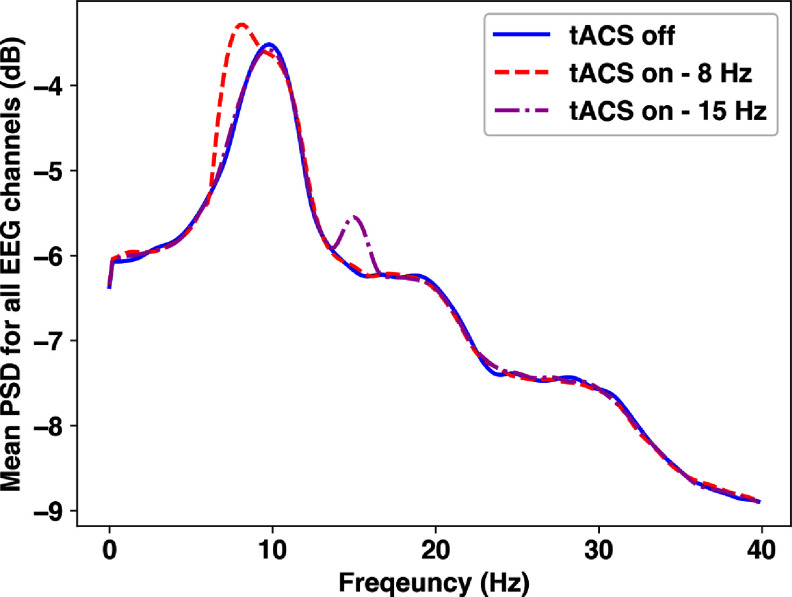
Average power spectral density across all EEG electrodes in the absence of stimulation (blue line) and with transcranial alternating current stimulation applied to P7-P8 at 8 Hz (red line) and 15 Hz (magenta line).

**Figure 9.**
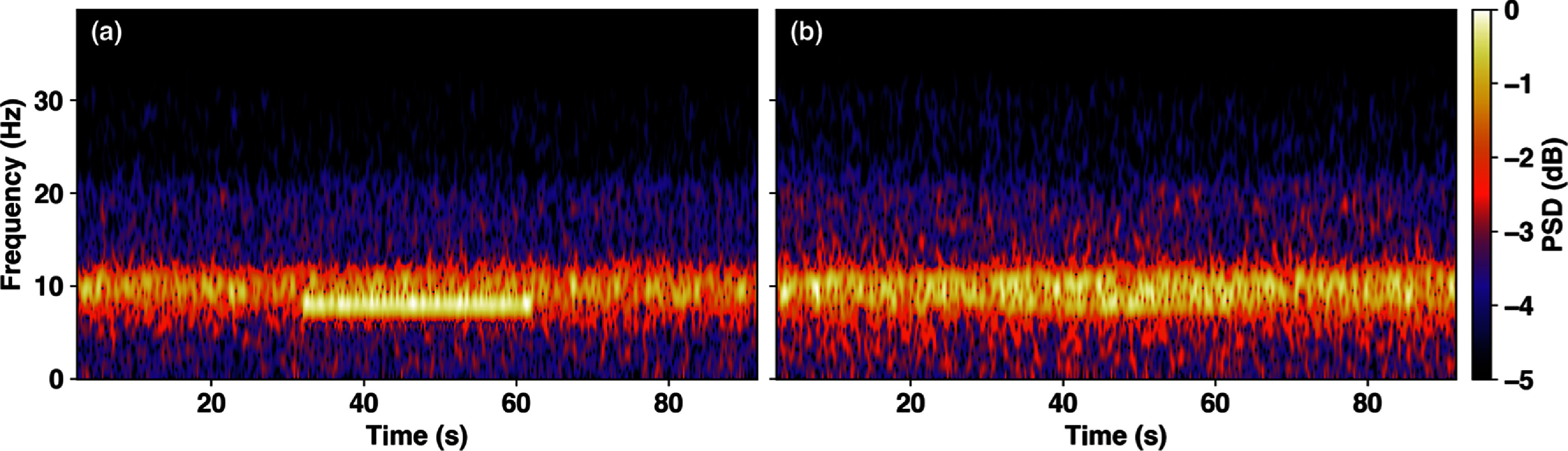
Spectrogram of activity at (a) P7 and (b) AF7 during the 8 Hz stimulation applied to P7-P8 from 31 to 61 s. Stimulation enhances the power spectral density around its fundamental frequency during the application interval. As shown, the strength of this effect is influenced by the spatial distribution of stimulation. In this case, P7 is a channel close to the stimulation electrodes, while AF7 is more distant. Consequently, the stimulation has a larger impact on P7 in comparison to AF7.

**Figure 10.**
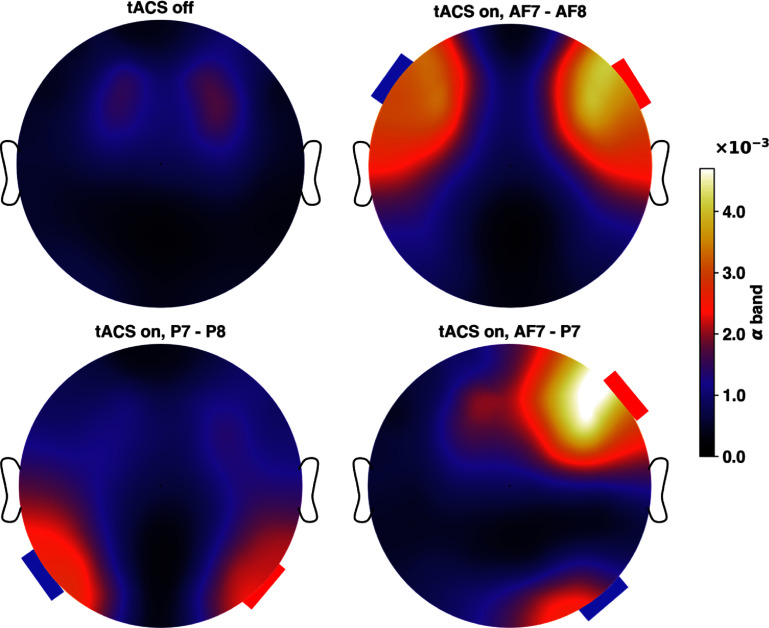
Topographic representation of the *α*-band (8–12 Hz) EEG-related power spectral density distribution before and during transcranial alternating current stimulation with three different stimulation configurations: P7-P8, P7-AF7, and AF7-AF8, all at a frequency of 8 Hz. As shown, the PSD distribution aligns with the induced E-field distribution during stimulation. This highlights the spatial specificity of stimulation-induced modulation in the *α*-band.

As expected, tACS increases the EEG power spectral density (PSD) around the stimulation frequency (see figure 8), consistent with findings in the two-column Jansen-Rit model in [104]. Figure 8 shows the average PSD for all EEG channels for 8 Hz and 15 Hz stimulation applied to P7-P8, as compared to baseline. The corresponding spectrograms in figure 9) also show the stimulation frequency-related PSD increase during the stimulation interval. To visualize the spatial distribution of the effects of stimulation (topographic maps in figure 10), we investigated the *α*-band EEG spectrum (from 8 to 12 Hz) when 8 Hz stimulation was applied to P7-P8, P7-AF7, or AF7-AF8. As anticipated, increases in *α*-band activity are most prominent near the electrodes, where the field magnitudes are highest.

## 4. Discussion

In this study, we developed an automated pipeline for personalized NIBS modeling for two primary purposes: (i) the optimization of NIBS parameters to achieve targeted exposures, and (ii) the investigation of the impacts of NIBS on whole brain dynamics. The developed pipeline achieves these goals, facilitating the optimization of NIBS parameters with an eye towards precise control of brain dynamics in an impact-driven manner.

Our pipeline incorporates a novel AI-based tool for generating personalized 3D head models using subject-specific anatomical imaging data. The tool demonstrates the capability to differentiate up to 40 tissues using T1-weighted MRI, surpassing other leading segmentation tools, such as *charm* [88] and *headreco* [13], in terms of detail, accuracy, and speed for most tissues (see section 3.1). The new approach achieves excellent generalization performance due to extensive augmentation of the training data, which comprises a large, multi-vendor, multi-field strength dataset [105]. Furthermore, the AI segmentation tool is robust to local minima, thereby avoiding a common drawback of registration-based approaches, which may consequently fail on entire regions (e.g. neck or face).

Implementing the pipeline on the open, cloud-based *o^2^S^2^PARC* platform allows for flexible reuse and sharing of the various constituent modules. It also enables the realization and sharing of highly automated and user-friendly workflows, such as TIP, tailored for specialized applications.

The TIP tool was developed to support tTIS research (section 3.2). It offers a step-by-step guided workflow to identify optimal electrode placement strategies for selectively targeting specific brain regions (in terms of exposure), and supports interactive exposure exploration, configuration refinement, and weighing of multiple optimization goals. TIP provides a user-friendly environment to aid clinicians with limited expertise in computational modeling in the development of optimized protocols for planned or ongoing studies.

Personalization is supported on multiple levels, including anatomical geometry, tissue property assignment, network connectivity, EM exposure, and LF matrices, leveraging the increased accessibility of rich and high quality imaging data. Using tTIS as a test case, we demonstrated that both the selection of model creation algorithm, and the use of personalized anatomical models (anatomy and dielectric properties) per se, significantly influence exposure predictions.

Regulators and standards distinguish verification (ensuring that a model implements the desired behavior) and validation (ensuring that the intended behavior reproduces the relevant aspects of the real-world one) [106, 107]. This study provides (respectively references) verificatory evidence for the segmentation and EM simulation through comparison with independently generated reference solutions. However, validation is complicated by the limited direct access to the predicted quantities (local field, local neural activity, etc) *in vivo*—particularly with regard to neural dynamics, where most measurements provide indirect (e.g. behavioral) or coarse-grained (e.g. EEG, fMRI) data. Our exposure simulations have received a degree of validatory confirmation through sparse measurements in cadavers [108] and epilepsy patients with implanted SEEG electrodes [109].

Application of the overall pipeline and its individual modules has been demonstrated in this study through a range of illustrative examples (such as assessing the impact of tACS on brain dynamics and EEG signals), producing results that qualitatively conform to expected brain behavior/responses. Using the presented pipeline, we have ourselves observed qualitative correlations between inter-subject differences in simulated exposure and measured brain activity (fMRI) and behavioral responses (learning) reported in [91, 110], as well as between graph theoretical metrics of simulated network dynamics of recovering stroke patients and symptom severity, though these early results are sensitive to modeling choices and require careful analysis in a separate study. Studies have shown that TVB models are capable of localizing epileptic foci in a personalized manner, superior to SEEG localization [48, 111]. However, additional investigations are necessary to better understand the strengths and limitations of our modeling approach and pipeline in terms of reproducing and predicting relevant features of brain activity at a single subject level. Such understanding will be required prior to addressing the key questions of whether applying personalized modeling results in improved therapeutic outcomes and how to apply it.

Moreover, application of the pipeline in a clinical setting demands a practical consideration of time and resource constraints, and while the computational demands of the pipeline are non-negligible (on the order of hours; dominated by data preprocessing, SC extraction, and EM simulations), these steps are fully automated and performed only once per subject. This facilitates compatibility with clinical applications, such as treatment planning, where model preparation can be completed in advance of an intervention. To further improve efficiency and move toward real-time applications, we are pursuing several complementary approaches: (i) cloud deployment enables parallel execution of computationally intensive tasks, while specialized acceleration strategies, such as a boundary element method solver amenable to lower–upper (LU) decomposition, physics-informed neural networks (PINNs), reduced-order modeling for electro placement optimization, and graphical processing unit (GPU)-accelerated computing, are being developed to expedite demanding and/or repeated calculations; (ii) for real-time applications such as closed-loop control, we are investigating surrogate modeling (SuMo) schemes to create efficient predictors using strategically selected simulation results (feedback during operation can help compensate for model inaccuracies); and (iii) we are exploring model simplification strategies, including NMM lattice coarse-graining and hybrid region/surface-based approaches, to identify acceptable trade-offs between computational efficiency and model fidelity.

Nevertheless, several limitations necessitating further development and/or research can already be identified:

• The AI-based anatomical model generation has been trained on a large T1 MRI multi-vendor, multi-field strength dataset (CC359) with strong augmentation to increase segmentation robustness. However, these datasets exclusively cover healthy adults. They do not include prominent pathological features, such as broken bones, tumors, or brain lesions. The latter is particularly relevant when modeling stroke patients with large liquified lesions (known to influence current pathways [112]), which are also likely to affect DTI-based connectivity quantification and tissue property assignment. The segmentation performance on image data from children has not been quantified and might be inferior. Fine-tuning or retraining to include infants and/or disease classes (lesions, tumors, liquid) could help;
• The reliability of extracting SC from DWI is sensitive to various factors, including motion artifacts, susceptibility-induced distortions, and eddy current effects, all of which can introduce significant errors into the data, warranting caution when interpreting the results [113]. To address this shortcoming, personalized SC may be combined with well-validated template-based approaches to enhance robustness and generalizability. This hybrid approach leverages the strengths of subject-specific information while benefiting from the stability of template-based methods;
• NMMs typically involve a large number of parameters. In this work, the Jansen-Rit model parameters were mostly set to default values (table 1), and SC information was used to personalize long-range connectivity. However, it is generally desirable to tune parameters, such that the overall model captures the relevant features (e.g. PSD, FC matrices, and DFC) of measured signals such as EEG, fMRI, etc. For epilepsy in particular, parameter tuning is a highly active field of research (e.g. [114–116]). Future iterations of the pipeline will incorporate dedicated parameter tuning modules;
• Sub-cortical regions were modeled in the same fashion as cortex, which is a common, but highly oversimplified approach. Instead, they likely require specialized NMMs or multi-scale approaches using detailed SNNs. The latter approach has recently garnered attention for its promise in DBS applications [64];
• The current pipeline was developed with EM NIBS techniques in mind. However, it may also be extended to support invasive forms of stimulation, and non-EM stimulation (e.g. low-intensity focused ultrasound (LIFUS); see [117] for a tentative approach). Furthermore, while the $\lambda\cdot E$ coupling term is useful for tACS and tDCS applications, an optimal coupling model remains to be determined for tTIS, LIFUS and other brain stimulation methodologies whose underlying mechanisms are less well established. Regarding recording modalities, future iterations of the pipeline will move beyond EEG signal modeling to support SEEG, fMRI, and positron emission tomography (PET) etc;
• While systematic uncertainty quantification of NIBS EM exposure modeling has been performed [118], covering diverse factors, such as tissue property variability and uncertainty, electrode placement uncertainty, and numerical errors (discretization, solver convergence), it is currently unclear, how a comparable analysis of the overall pipeline could be realized, especially in what concerns the models used for modeling neural dynamics. In view of the highly oversimplified nature of the brain network, the analysis will likely have to limit itself to the aspect of how successful it is in predicting relevant characteristics of brain activity and their inter-subject differences within a given context-of-use, and whether it improves stimulation effectivity;
• The pipeline currently examines short-term NIBS effects, i.e. modified brain dynamics during stimulation. However, the incorporation of plasticity models will be necessary to investigate the long-term effects of stimulation. Preliminary efforts in that direction have already been initiated—see, e.g. Giannakakis *et al* [60], who introduced a simple form of Hebbian plasticity in a Wilson–Cowan NMM;
• The Jansen-Rit NMM was primarily developed to reproduce realistic EEG signals, but may not be optimal for modeling overall brain network dynamics. However, a key advantage of TVB lies in its extensive array of sophisticated NMMs, facilitating future pipeline-based investigations employing alternative models.

A number of ethical aspects also require consideration: Personalization necessarily entails the use of privacy-sensitive individual data and can also result in the creation of models with identifying information. Thus, privacy protection is required, especially given the online accessible nature of our pipeline. In addition to o^2^S^2^PARC’s in-built security features (AWS inherent security, automatic source code scanning for known security issues, open-source transparency, micro-service and API design, expiring API keys, multi-tenant platform, two-factor authentication, enforced registration), privacy-oriented measures include the ability to deploy o^2^S^2^PARC locally (e.g. protected hospital infrastructure) or to perform sensitive steps locally, while only uploading anonymized data for online pipeline execution. The latter may be automated, e.g. with a preprocessing executable that automatically constructs the anatomical model, then defaces the results (and original image-data) before uploading these to o^2^S^2^PARC. Ethical questions pertinent to behavior modifying brain stimulation might also be amplified by the proposed shift to closed-loop control of high-level brain state metrics.

## 5. Conclusions

In this study, a highly automated, modular, and extendable pipeline for personalized NIBS modeling was developed on o^2^S^2^PARC—an open, cloud-based infrastructure for collaborative, FAIR, and reproducible computational life science—to improve the understanding of healthy and pathological brain dynamics, and to develop optimized, patient-specific stimulation strategies. It comprises the following steps:

• Personalized 3D anatomical models: The pipeline begins by harnessing an AI-based method to generate MRI-derived, personalized 3D anatomical models. Comparison with commonly employed tools demonstrates the superior quality of our algorithm in terms of accuracy, detail, performance, and robustness;
• Personalized dielectric properties: Drawing on DWI data, the pipeline assigns heterogeneous, anisotropic dielectric property maps;
• Personalized brain SC: DWI data is used to extract SC information for personalized brain network model generation;
• EM simulations: The pipeline employs Sim4Life to (i) simulate EM neurostimulation exposures and (ii) compute LF matrices that relate brain activity to measurable EEG signals in a personalized manner;
• Exposure optimization: Stimulation parameters are optimized with regard to multiple stimulation quality-relevant metrics (targeting strength, selectivity, collateral stimulation). This optimization is a key feature in tailoring neurostimulation therapies to individual subjects and maximizing efficacy;
• Coupling induced E-fields to TVB-based WBMs: The induced E-fields are coupled to TVB-based WBMs, permitting the consideration of the therapeutically relevant impacts on brain network activity;
• Prediction of measurable signals (e.g. EEG): Utilizing the reciprocity theorem and LF matrices, the pipeline predicts measurable signals (here, EEG) in a personalized manner, facilitating the interpretation of, and validation against measurable signals;
• Brain dynamics analysis using DFC: The pipeline concludes by supporting an in-depth analysis of simulated and measured brain activity, leveraging graph theoretical and DFC approaches to characterize overall brain states and dynamics.

Furthermore, utilizing the developed modules (see section 2), TIP—a tTIS planning tool—was developed to support research groups with limited modeling expertise in deriving optimal stimulation parameters for targeted and selective tTIS. Free access for scientific use is available through registration at https://tip.itis.swiss/.

The current work is part of an ongoing effort to advance beyond pure exposure optimization towards physiological response-/impact-driven optimization. While exposure optimization is increasingly well-established and reliable, the optimization of physiological responses is subject to a range of uncertainties (not merely in parameter values, but also regarding the model form itself). However, impact-centric modeling has the benefit of assessing and optimizing quantities directly relevant to stimulation safety and efficacy.

As a proof-of-concept, we used the pipeline to simulate a tACS application using imaging data from a healthy subject. The results demonstrate plausible changes, e.g. stimulation-dependent shifts in the spatiotemporal EEG PSD. High-level brain dynamics were studied in terms of functional network correlation metrics, revealing stereotyped transition dynamics between synchonized and desynhronized hemisphere states (see figure 5).

Our ultimate vision is to develop an advanced system that integrates (i) model-based, closed-loop control (ii) leveraging hybrid simulations of EM fields and brain network dynamics (iii) combined with real-time feedback from sources such as EEG to (iv) generate spatiotemporal stimulation strategies that (v) drive metrics characterizing high-level brain state (e.g. fluidity) towards values observed in healthy populations (or more generally, towards a targeted brain state). The central hypothesis is that normalization of these metrics will confer therapeutic benefits. By formulating optimization goals in terms of high-level brain state metrics, rather than the activation of a target brain region, we account for the interdependent nature of brain circuits, where modulation of one node can influence the entire network. This strategy is particularly advantageous in pathological conditions where restoring local function may be infeasible due to irreversible brain tissue damage/alterations, but global dynamic state normalization remains possible. Such an approach will require (i) the creation and tuning of a personalized model that is representative of the underlying nonlinear behavior of the brain and (ii) the identification of sub-states of activity that are characteristic of the individual brain dynamics and establish a relationship between function and structure. Using the information obtained with this model, (iii) an efficient SuMo can be generated, which relates stimulation parameters (drivers) to their predicted impact on both measurable quantities (observables) and simulated response metrics (objectives; these should be chosen to be tightly related to the intended stimulation impact). Such a SuMo can be used as interior-model (see [119–121]) for (iv) a model predictive control strategy that—based on the difference between measured and predicted observables—identifies dynamic drivers that optimize the evolution of the objective up to a time-horizon [119]. Potentially, (v) the SuMo can be iteratively improved by back-propagating observed differences between the evolution of predicted and measured observables. Finally, the inclusion of plasticity modeling will enable the study of therapeutic interventions aimed at long-term network reorganization, instead of focusing solely on the immediate effects of stimulation.

## Data Availability

The data that support the findings of this study will be openly available following an embargo at the following URL/DOI: https://zenodo.org/records/8252 501. Data will be available from 01 April 2025.
